# A comparison of approaches for combining predictive markers for personalised treatment recommendations

**DOI:** 10.1186/s13063-020-04901-2

**Published:** 2021-01-06

**Authors:** Matthias Pierce, Richard Emsley

**Affiliations:** 1 Centre for Biostatistics, School of Health Sciences, Faculty of Biology, Medicine and Health, University of Manchester, Manchester Academic Health Science Centre, 1st Floor, Jean McFarlane Building, Oxford Road, Manchester, M13 9PL UK; 2 Department of Biostatistics and Health Informatics, Institute of Psychiatry, Psychology and Neuroscience, King’s College London, London, UK

**Keywords:** Personalised medicine, Stratified medicine, Precision medicine, Personalised treatment recommendations, Predictive biomarkers, Moderators

## Abstract

**Background:**

In the presence of heterogeneous treatment effects, it is desirable to divide patients into subgroups based on their expected response to treatment. This is formalised via a personalised treatment recommendation: an algorithm that uses biomarker measurements to select treatments. It could be that multiple, rather than single, biomarkers better predict these subgroups. However, finding the optimal combination of multiple biomarkers can be a difficult prediction problem.

**Methods:**

We described three parametric methods for finding the optimal combination of biomarkers in a personalised treatment recommendation, using randomised trial data: a regression approach that models outcome using treatment by biomarker interactions; an approach proposed by Kraemer that forms a combined measure from individual biomarker weights, calculated on all treated and control pairs; and a novel modification of Kraemer’s approach that utilises a prognostic score to sample matched treated and control subjects. Using Monte Carlo simulations under multiple data-generating models, we compare these approaches and draw conclusions based on a measure of improvement under a personalised treatment recommendation compared to a standard treatment. The three methods are applied to data from a randomised trial of home-delivered pragmatic rehabilitation versus treatment as usual for patients with chronic fatigue syndrome (the FINE trial). Prior analysis of this data indicated some treatment effect heterogeneity from multiple, correlated biomarkers.

**Results:**

The regression approach outperformed Kraemer’s approach across all data-generating scenarios. The modification of Kraemer’s approach leads to improved treatment recommendations, except in the case where there was a strong unobserved prognostic biomarker. In the FINE example, the regression method indicated a weak improvement under its personalised treatment recommendation algorithm.

**Conclusions:**

The method proposed by Kraemer does not perform better than a regression approach for combining multiple biomarkers. All methods are sensitive to misspecification of the parametric models.

## Background

One of the primary aims of the modern paradigm of stratified medicine is to move beyond a one-size-fits-all approach that allocates treatment based on population average responses, towards identifying patient subgroups for whom a given treatment is beneficial and those for whom it is not. Given a patient population with heterogeneous treatment response, it might be possible to produce an algorithm for clinical use that provides a recommendation for treatment based on measurable traits (biomarkers). For these purposes, it is necessary to separate biomarkers into those that predict treatment response (moderating biomarkers) and those that predict the outcome, regardless of treatment (prognostic biomarkers). When the treatment choice is binary (the situation considered in this paper), the algorithm may recommend a treatment over an alternative for values of a single moderating biomarker, or a weighted combination of multiple moderating biomarkers. Such an algorithm is referred to as a personalised treatment recommendation (PTR).

In many disease areas, it might be that a combination of multiple biomarkers is more effective in identifying subgroups with a beneficial treatment outcome than any single biomarker [1]. Finding the optimal combination of biomarkers in a PTR algorithm is a challenging prediction problem. In order to avoid the confounding between treatment assignment and outcome, it is considered optimal that PTR’s are estimated from randomised controlled trial (RCT) data. A method used to estimate a PTR is to fit a regression model with treatment by biomarker interaction terms [2, 3]. Kraemer [4] proposes an alternative method that uses a parametric model fitted to all pairwise combinations of treated and control subjects. The method assigns a weight to each moderator from the correlation between pair’s average moderator value and the difference in their outcomes. A composite moderator is then derived from the individual moderator weights and used to derive a PTR.

In this paper, we expand the Kraemer method by utilising a prognostic score to sample matched pairs of treated and control subjects, rather than using all pairwise combinations. This prognostic score is the estimated treatment-free outcome in all subjects, regardless of treatment assignment. We use Monte Carlo simulations to compare the Kraemer method, its proposed modification and the regression method with respect to estimating a PTR. Their respective utility is measured using an estimate of the expected change in outcome under the PTR compared to a non-stratified approach. The comparisons are made under a range of data-generating mechanisms and sample sizes. The methods are then applied to data from a randomised controlled trial of pragmatic rehabilitation versus treatment as usual, for patients with chronic fatigue syndrome [5].

### Methods Constructing a personalised treatment recommendation

Following a randomised controlled trial, for a continuous outcome *Y* and binary treatment *A* (following Kraemer, treatment is effect-coded as +1/2 for treated and –1/2 for control), the outcome for the *i*th subject might be described by the linear model:

1 $$ {Y}_i={\alpha}_0+\alpha {X}_i+{A}_i\left({\beta}_0+\beta {Z}_i\right)+{e}_i $$

where *α* represents parameters for the *prognostic* variables in vector *X* (determining treatment-free outcome) and *β* are parameters for the *moderator* variables in vector *Z* (determining treatment effect heterogeneity). *β*_0_ represents the effect of treatment at the average value of the moderators.

### Kraemer method for creating a ‘composite moderator’

In order to estimate the overall moderating effect of multiple biomarkers, Kraemer proposes a method for creating a ‘composite moderator’ from a weighted sum of moderating biomarkers [4, 6]. In the first step, a dataset is constructed from all possible pairwise combinations of treated and untreated subjects. From the parameterisation in Eq. 1, the change in outcome between the *j*th pair of treated (superscript *T*) and control (superscript *C*) subjects is equivalent to:

$$ {Y}_j^T-{Y}_j^C=\alpha {\left({X}_j^T-{X}_j^C\right)}^{\intercal }+{\beta}_0+\beta {\left(\frac{Z_j^T+{Z}_j^C}{2}\right)}^{\intercal }+\left({e}_j^T-{e}_j^C\right) $$

which can be re-expressed as:

$$ \Delta  \left({Y}_j\right)=\alpha \Delta  \left({X}_j\right)+{\beta}_0+\beta \mu \left({Z}_j\right)+\Delta  \left({e}_j\right) $$

where *∆* represents the within-pair difference and *μ* represents the within-pair mean. Intuitively, if a single variable *Z* had a strong moderating effect, then we would expect the within-pair difference *∆*(*Y*_*j*_) would increase in line with the average value of the moderator. Similarly, if a single variable *X* had a strong prognostic effect, then we would expect that large within-pair differences in that variable would result in large within-pair differences in the outcome. Kraemer formally shows that, for a single marker, the moderator effect size is characterised by the correlation between *∆*(*Y*) and *μ*(*Z*) and the prognostic effect size is characterised by the correlation between *∆*(*Y*) and *∆*(*X*).

We can account for multiple, correlated moderating variables by regressing the average moderator value for *K* variables on the change in outcome $\Delta  (Y)={\sum}_1^K{w}_k\mu (Z)$ to calculate moderator-specific weights *w*_1_, *w*_2_, …*w*_*k*_. A combined moderator is obtained by the sum of the individual moderator weights: ${Z_i}^{\ast }=\sum \limits_1^K{w}_k{Z}_{ik}$. This combined moderator can be used to separate subgroups based on their expected treatment response (see below) [6].

### Modified Kraemer approach: matching on a prognostic score

We modify Kraemer’s approach to determine whether considering only treated and control pairs that have similar values of a ‘prognostic score’ improves its performance. A prognostic score is an estimate of the treatment-free outcome. This is calculated by fitting a regression model to the subjects in the control group only (thus excluding any moderators):

$$ \mu \left(Y,A=-1/2\right)={\alpha}_0+\alpha {X}^{\intercal } $$

The resulting model can be used to predict the outcome under the control condition, for subjects in both the control and treatment groups.

The resulting estimates are referred to as *prognostic scores* and have been used in observational research to control for confounding [7].

We propose modifying Kraemer’s approach so that the composite moderator is derived using a sample of treated and control pairs that only have similar values of their prognostic scores. In our implementation, we use a single nearest neighbour matching algorithm, with replacement, and with a calliper such that, for each treated subject, a control is sought that is within 0.1 times the standard deviation of the prognostic score. The calliper width is arbitrary and was set prior to any implementation.

The rationale for this modification is to minimise the contribution to *∆*(*Y*) by variables that are irrelevant to treatment effect modification. There is a trade-off between minimising this variance and losing treated and control pairs who do not fit the matching criteria. We investigate whether this modification results in an improvement on the Kraemer approach by applying these techniques to simulated datasets. First, we establish how to measure improvement in the context of determining a personalised treatment recommendation.

### Constructing a personalised treatment recommendation (PTR)

A PTR uses biomarker values to recommend whether a patient should be treated or not. For example, statins are recommended in the UK if a person is aged over 40 and if their estimated CVD risk is at least 10% over a 10-year period [8]. Formally, this can be represented as: $\mathrm{PTR}=\mathbbm{I}\left\{\mathrm{age}\ge 40\&\mathrm{CVD}\ \mathrm{risk}\ge 10\%\right\}$, where $\mathbbm{I}$ indicates that treatment (statins) is recommended over the alternative (no statins) when the bracketed expression is true.

Assuming higher values of a continuous outcome are advantageous, an optimal PTR is one that recommends treatment when, conditional on a set of moderating biomarkers *Z*, the mean outcome under treatment *μ*(*A* = 1/2, *Z*) is greater than the mean outcome under control *μ*(*A* =  − 1/2, *Z*):

$$ \mathrm{PTR}=\mathbbm{I}\left\{\mu \left(A=1/2,Z\right)-\mu \left(A=-1/2,Z\right)>0\right\} $$

Under the parametrisation in Eq. (1):

2 $$ \mathbbm{I}\left\{\mu \left(A=1/2,Z\right)-\mu \left(A=-1/2,Z\right)>0\right\}=\mathbbm{I}\left\{{\beta}_0+\beta {Z}^{\intercal }>0\right\} $$

The parameters *β*_0_ and *β* can be estimated using ordinary least squares regression with treatment by moderator interaction terms [2, 3]. This we refer to as the *regression approach*.

A PTR can be constructed using the Kraemer or modified Kraemer method using the following steps: (1) calculate the combined moderator; (2) regress the outcome on a model with treatment, the combined moderator and their interaction with treatment: $\mu \left(A,{Z}^{\ast}\right)={\alpha}_0+{\alpha Z}^{\ast }+A\left({\beta}_0^{\ast }+{\beta}^{\ast }{Z}^{\ast}\right)$ (where *Z*^∗^ is the combined moderator and ${\beta}_0^{\ast }+{\beta}^{\ast }$ are the average effect of treatment and the moderated effect, respectively); and (3) use these parameters to calculate the PTR: $\mathrm{PTR}=\mathbbm{I}\left\{\left({\beta}_0^{\ast }+{\beta}^{\ast }{Z}^{\ast}\right)>0\right\}$.

### Measuring the performance of a personalised treatment recommendations

Under randomisation, an unbiased estimate of the population mean outcome under a PTR is provided by the mean of the observed outcome in those receiving the treatment they were recommended weighted by the probability of being randomised to their respective group (π) [9–11]:

$$ \mu \left\{ PTR\right\}=\frac{1}{n}{\sum}^n\left(\frac{\left(A+1/2\right)\cdotp \left( PTR+1/2\right)}{\pi }Y+\frac{\left(1/2-A\right)\cdotp \left(1/2- PTR\right)}{1-\pi }Y\right) $$

This can be contrasted with the average outcome under treatment $\mu \left\{A=1/2\right\}=\frac{1}{n}{\sum}^n\left(\frac{A+1/2}{\pi }Y\right)$ or control $\mu \left\{A=-1/2\right\}=\frac{1}{n}{\sum}^n\left(\frac{1/2-A}{1-\pi }Y\right)$ to get the parameters: *θ*_*T*_ = *μ*{*PTR*} − *μ*{*A* = 1/2 } and *θ*_*C*_ = *μ*{*PTR*} − *μ*{*A* =  − 1/2 }. These are interpreted as the expected change in outcome under a PTR compared to a policy where everybody receives treatment or everybody receives control.

In a simulation study, an additional measure of the performance of a PTR is the rate of misclassification; that is, the proportion of subjects whose PTR conflicts with their known optimal treatment: *ℙ*(PTR(*X*) ≠ PTR^opt^), where PTR^opt^ indicates treatment if the simulated outcome under treatment is greater than the simulated outcome under control.

### Simulations comparing approaches

Monte Carlo simulations were constructed to compare the regression, Kraemer and modified Kraemer approaches to estimating a PTR. Training datasets were simulated with sample sizes 75, 200 and 300 with 1:1 randomisation. These datasets were generated under a range of scenarios (shown in Table 1). PTRs were estimated using all three approaches and applied to a test dataset of the same size and generated using the same specifications as the training dataset. For each PTR, we use the test dataset to calculate the change under PTR (*θ*_*T*_) and the misclassification rate. For each data-generating scenario, 5000 simulations were carried out and we evaluate each method by averaging *θ*_*T*_ and the misclassification across simulations.

**Table 1:** Different scenarios for simulations, comparing approaches to combining multiple biomarkers to construct personalised treatment recommendations

#	Scenario	Data generation model	Variables in the prediction model
1.	Simple linear	*Y* = 3*X*_1_ + 2*Z*_1_ − 0.5*Z*_3. *lv*2_ − 1.5*Z*_3. *lv*3_ +*A*(2 + 2*Z*_1_ − 1.5*Z*_2_ − 3*Z*_3. *lv*3_) + *e*	All variables
2.	Linear with weak moderators	*Y* = 3*X*_1_ + 2*Z*_1_ − 0.5*Z*_3. *lv*2_ − 1.5*Z*_3. *lv*3_ +*A*(2 + 0.5*Z*_1_ − 0.5*Z*_2_ − 0.25*Z*_3. *lv*3_) + *e*	All variables
3.	Strong unobserved prognostic marker	*Y* = 12*U*_1_ + 3*X*_1_ + 2*Z*_1_ − 0.5*Z*_3. *lv*2_ − 1.5*Z*_3. *lv*3_ +*A*(2 + 2*Z*_1_ − 1.5*Z*_2_ − 3*Z*_3. *lv*3_) + *e*	Excluding *U*_1_
4.	Strong unobserved moderator variables	*Y* = 3*X*_1_ + 2*Z*_1_ − 0.5*Z*_3. *lv*2_ − 1.5*Z*_3. *lv*3_ + *A*(2 + 10*U*_2_ + 2*Z*_1_ − 1.5*Z*_2_ − 3*Z*_3. *lv*3_) + *e*	Excluding *U*_2_
5.	Misspecified prognostic part	*Y* = 3*X*_1_ + 2*Z*_1_ − 0.5*Z*_3. *lv*2_ − 1.5*Z*_3. *lv*3_ + 2*X*_1_^2^ + 1.5*U*_1_*X*_1_ − 1.5*X*_1_*M*_1_ + 0.5*X*_1_*Z*_1_*Z*_3. *lv*3_ +*A*(2 + 2*Z*_1_ − 1.5*Z*_2_ − 3*Z*_3. *lv*3_) + *e*	All linear terms, excluding *U*_1_
6.	Misspecified moderator part	*Y* = 3*X*_1_ + 2*Z*_1_ − 0.5*Z*_3. *lv*2_ − 1.5*Z*_3. *lv*3_ +*A*(2 + 2*Z*_1_ − 1.5*Z*_2_ − 3*Z*_3. *lv*3_ + 2*Z*_1_*Z*_2_ − 1.5*Z*_1_*Z*_2_*U*_2_ + 0.5*Z*_1_*Z*_2_*Z*_3. *lv*2_) + *e*	All linear terms, excluding *U*_2_
7.	Non-linear model	*Y* = (3*X*_1_ + 2*Z*_1_ − 0.5*Z*_3. *lv*2_ − 1.5*Z*_3. *lv*3_) +*A*(2 + 2*Z*_1_ − 1.5*Z*_2_ − 3*Z*_3. *lv*3_)/(−2*Z*_1_ + 1.5*Z*_2_ − 1.5*Z*_3. *lv*3_) + *e*	All as linear terms

### Application to randomised trial data

The three approaches to constructing a PTR were applied to data from the Fatigue Intervention Nurses Evaluation (FINE) randomised trial [12]. This trial randomised 296 patients diagnosed with Chronic Fatigue Syndrome to three groups: home-delivered pragmatic rehabilitation, supportive listening or treatment as usual. It found marginal evidence that home-delivered pragmatic rehabilitation reduced fatigue scores, compared to treatment as usual (effect estimate − 1.18, 95% confidence interval − 2.18 to − 0.18; 2-sided *p* value = 0.021) [5]. An exploratory secondary analysis considered individual moderators of this effect for those randomised to either pragmatic rehabilitation or treatment as usual (*n* = 195) using variables collected at baseline [13]. Here, we combine these moderators to identify whether a PTR that recommends treatment to a subset improves on a scenario where everybody receives treatment-as-usual approach.

The original effect estimate was not enough to change policy, given that the treatment was costly. Stratifying the treatment so that it is provided to those who benefit most might be more cost-effective than providing treatment to everybody. For our analysis, variables were designated prognostic (in the regression or modified Kraemer approaches) if they were identified as such in the initial analysis [13]. The outcome variable was change in fatigue score from baseline to follow-up (with positive values indicating an improvement). Variables were designated moderators if they had a univariate *p* value in the initial analysis of less than 0.10. Moderating variables were then excluded then if their *p* value was greater than 0.3, in a multivariate model that included all moderating variables. Three variables remained to include in the PTR: baseline fatigue score (*p* value for interaction = 0.24), EQ-5D mobility (no problems, some problems, severe problems, *p* = 0.16) and score on the Oslo Social Support scale, relating to concern (*p* = 0.15). The data was randomly split in half between training and test datasets (size *n* = 98 and *n* = 97 respectively) and then PTRs were evaluated on the test dataset using the parameter *θ*_*C*_ outlined above. Inference for this parameter was determined by drawing 1000 bootstrap samples and using the normal approximation.

## Results

### Simulations

Across all data-generating scenarios and sample sizes, the regression method was superior to both the Kraemer and the modified Kraemer methods: on average, it was both associated with higher values of *θ*_*T*_ (the expected benefit under PTR compared to treating everyone) and the lowest misclassification rate (Table 2). The modification of the Kraemer method, where treated and control subjects are matched on their prognostic score, improved on the Kraemer method across most data-generating scenarios. The exception is the scenario with a strong prognostic variable that is not included in the regression model. This suggests that the prognostic score is useful only when it captures sufficient variation in the prognostic effects. Post hoc, we changed the size of the calliper distance but this did not make any notifiable difference until it was < 0.05 SD or > 1.5 SD of the prognostic score (Table 2).

**Table 2:** Results of simulation studies showing mean values of theta and misclassification rate across 5000 simulations under a range of data-generating scenarios

Scenario	Sample size	Parameter	Method		
			Regression	Kraemer	Modified Kraemer
1a. Simple linear	50	*θ* (SD)	0.72 (0.676)	0.46 (0.810)	0.62 (0.679)
		Misclass. rate	0.05	0.21	0.14
	200	*θ* (SD)	0.71 (0.473)	0.58 (0.555)	0.68 (0.468)
		Misclass. rate	0.04	0.15	0.09
	300	*θ* (SD)	0.72 (0.337)	0.65 (0.397)	0.70 (0.335)
		Misclass. rate	0.02	0.10	0.06
1b. Simple linear with correlated biomarkers	50	*θ* (SD)	0.73 (0.629)	0.50 (0.695)	0.59 (0.616)
		Misclass. rate	0.05	0.21	0.17
	200	*θ* (SD)	0.61 (0.422)	0.48 (0.483)	0.56 (0.423)
		Misclass. rate	0.05	0.17	0.11
	300	*θ* (SD)	0.62 (0.276)	0.57 (0.331)	0.61 (0.278)
		Misclass. rate	0.02	0.1	0.06
2. Linear with weak moderators	50	*θ* (SD)	0.71 (0.665)	0.45 (0.799)	0.61 (0.667)
		Misclass. rate	0.05	0.21	0.13
	200	*θ* (SD)	0.71 (0.482)	0.56 (0.567)	0.66 (0.481)
		Misclass. rate	0.04	0.15	0.09
	300	*θ* (SD)	0.72 (0.336)	0.66 (0.401)	0.71 (0.336)
		Misclass. rate	0.02	0.1	0.06
3. Strong unobserved prognostic marker	50	*θ* (SD)	− 0.23 (3.150)	− 0.24 (3.149)	− 0.31 (3.115)
		Misclass. rate	0.41	0.41	0.42
	200	*θ* (SD)	− 0.23 (3.150)	− 0.24 (3.149)	− 0.31 (3.115)
		Misclass. rate	0.41	0.41	0.42
	300	*θ* (SD)	0.07 (1.556)	0.06 (1.570)	0.01 (1.535)
		Misclass. rate	0.32	0.32	0.34
4. Strong unobserved moderator variables	50	*θ* (SD)	0.22 (1.328)	0.16 (1.405)	0.14 (1.323)
		Misclass. rate	0.42	0.45	0.45
	200	*θ* (SD)	0.43 (0.893)	0.35 (0.946)	0.35 (0.911)
		Misclass. rate	0.42	0.43	0.44
	300	*θ* (SD)	0.56 (0.606)	0.51 (0.654)	0.52 (0.620)
		Misclass. rate	0.41	0.42	0.42
5. Misspecified prognostic part	50	*θ* (SD)	0.53 (0.866)	0.34 (1.010)	0.40 (0.882)
		Misclass. rate	0.17	0.25	0.22
	200	*θ* (SD)	0.62 (0.582)	0.49 (0.682)	0.57 (0.588)
		Misclass. rate	0.12	0.18	0.15
	300	*θ* (SD)	0.67 (0.406)	0.60 (0.495)	0.65 (0.407)
		Misclass. rate	0.09	0.13	0.11
6. Misspecified moderator part	50	*θ* (SD)	0.70 (0.773)	0.44 (0.872)	0.58 (0.775)
		Misclass. rate	0.16	0.25	0.21
	200	*θ* (SD)	0.78 (0.559)	0.60 (0.651)	0.72 (0.558)
		Misclass. rate	0.13	0.2	0.16
	300	*θ* (SD)	0.80 (0.381)	0.72 (0.443)	0.77 (0.382)
		Misclass. rate	0.12	0.16	0.14
7. Non-linear model	50	*θ* (SD)	2.11 (8.414)	1.94 (8.606)	2.10 (8.566)
		Misclass. rate	0.45	0.45	0.44
	200	*θ* (SD)	2.89 (9.242)	2.84 (10.822)	1.62 (10.261)
		Misclass. rate	0.44	0.44	0.43
	300	*θ* (SD)	0.31 (10.085)	0.06 (10.831)	− 0.30 (10.797)
		Misclass. rate	0.44	0.44	0.43

In the scenario with a non-linear data-generating model, no method, on average, constructed a PTR where subjects had a better outcome compared to a policy where everybody was treated. It is worth noting that, in this scenario, if each simulated subject were allocated the treatment they should have received, then their expected outcome would be, on average, 1.40 higher than if everybody were treated.

### Trial data

The results of the PTR algorithms and the estimated change under a PTR, compared to a treatment-as-usual approach, are shown in Table 3. There is weak evidence that the regression method results in a PTR that results in a greater reduction in chronic fatigue symptoms compared to an approach where everybody receives treatment as usual (*θ* = 1.92, 95% CI − 0.65 to 4.49). There was little evidence that a PTR estimated using the Kraemer method or the modified Kraemer method results in an improvement compared to treatment as usual (*p* = 0.47 and *p* = 0.13 respectively). Eight subjects were excluded when implementing the modified Kraemer approach because they did not have a match within the set calliper distance of the prognostic score.

**Table 3:** Results of the analysis of FINE data

Method	PTR algorithm	*θ*	*p* value	95% CI
Regression	I(−2.020 + 1.709. baselinefat + 1.705. EQ5D + 1.685. concern < 0)	1.92	0.07	− 0.65 to 4.49
Kraemer	I(−2.342 + 0.633. baselinefat + 2.253. EQ5D + 2.069. concern < 0)	− 0.10	0.47	− 2.26 to 2.06
Modified Kraemer	I(−2.365 + 1.278. baselinefat + 1.568. EQ5D + 2.101. concern < 0)	0.69	0.13	− 0.49 to 1.87

## Discussion

This paper reported on a comparison of three methods for constructing personalised treatment recommendations from randomised controlled trial data: the regression method that models outcome using treatment by moderator biomarker interactions; a method proposed by Kraemer that forms a combined moderator from individual moderator weights, calculated on all treated and control pairs; and a modification of Kraemer’s approach that utilises a prognostic score to sample pairs of treated and control subjects. Across all simulations, the regression approach outperformed Kraemer’s approach. The modification of Kraemer’s approach appeared to indicate higher values of *θ*, except in the case where there was a strong unobserved prognostic marker. The superiority of the regression approach was replicated using real-world data from a randomised trial of home-delivered pragmatic rehabilitation for chronic fatigue patients; however, for this example, no method conclusively demonstrated that a PTR does better than a policy of ‘treatment as usual’ despite there being several individual moderators of treatment effect [13]. Therefore, in this case, we conclude that forming a PTR is more difficult than finding individual treatment effect moderators.

All three methods described here use linear parameterisation to model trial data, and therefore, the efficacy of these methods relies on the models being correctly specified. In many situations, non-linear models may be more applicable and when there are many variables then the likelihood of correctly specifying the model might be low. For example, none of the approaches, on average, indicated an improvement under PTR in the scenario with a non-linear data-generating model. These simulations were limited because they did not include any variable selection or transformations of variables based on model fit. Such processes require care and often require knowledge of the variables at hand. In practice, researchers will be insuring against model underfitting by testing for non-linear terms and higher-order interactions. Overfitting of these models could be counterbalanced using regularisation techniques, such as Lasso regression. Additionally, methods exist that are more robust to model misspecification, for example methods that seek to maximise the expected outcome under a PTR using classification techniques [14–16].

The application to the FINE randomised control trial showed that a PTR, as estimated using the regression approach, might result in an improvement over a recommendation where everybody is provided treatment as usual; however, the 95% confidence interval for all approaches included estimates where the PTR strategy is associated with a small amount of harm. These results should not be over-interpreted: the 95% confidence intervals were wide, which indicates insufficient power to detect a change. Data from another trial testing the use of the PTR would be needed to confirm whether any PTR results in an overall benefit. Another aspect that should be considered is whether including cost information in the measure of benefit has an effect on the decision to adopt a PTR strategy.

In the discussion to the paper, Kraemer says: ‘an irrelevant baseline factor and a non-specific predictor can have no influence on making different choices between [treatments]’. We argue that this appears to be false, based on our findings: information from a prognostic score appears to result in a composite moderator that more effectively discriminates between those who should receive treatment and those that should not. Whilst the Kraemer approach does not appear to improve on the regression approach, it should be noted that the original paper provides a useful example of how to judge the relative effect sizes of multiple modifiers that would form a useful exploratory analysis before forming a PTR.

## Conclusion

Our simulations demonstrate that the parametric method proposed by Kraemer does not result in a more effective personalised treatment recommendation than a method that uses a regression model. Utilising a prognostic score improves the Kraemer method, however not to an extent that it should be adopted over the regression method.

## Data Availability

Stata do-files used to construct simulations are available on request from the corresponding author.

## Abbreviations

PTR: Personalised treatment recommendation
RCT: Randomised controlled trial
FINE: Fatigue Intervention Nurses Evaluation randomised trial
